# A Fumonisins Immunosensor Based on Polyanilino-Carbon Nanotubes Doped with Palladium Telluride Quantum Dots

**DOI:** 10.3390/s150100529

**Published:** 2014-12-30

**Authors:** Milua Masikini, Stephen N. Mailu, Abebaw Tsegaye, Njagi Njomo, Kerileng M. Molapo, Chinwe O. Ikpo, Christopher Edozie Sunday, Candice Rassie, Lindsay Wilson, Priscilla G. L. Baker, Emmanuel I. Iwuoha

**Affiliations:** SensorLab, Department of Chemistry, University of the Western Cape, Robert Sobukwe Road, Bellville 7535, Cape Town, South Africa; E-Mails: masikinimilua@gmail.com (M.M.); smnzioki2007@gmail.com (S.N.M.); 3080361@myuwc.ac.za (A.T.); njaginjomo@gmail.com (N.N.); 3050950@myuwc.ac.za (K.M.M.); cikpo@uwc.ac.za (C.O.I.); csunday@uwc.ac.za (C.E.S.); 2734778@myuwc.ac.za (C.R.); 2724554@myuwc.ac.za (L.W.); pbaker@uwc.ac.za (P.G.L.B.)

**Keywords:** anti-fumonisins antibody, impedimetric immunosensor, palladium telluride quantum dots, poly(2,5-dimethoxyaniline)-multi-wall carbon nanotubes, electrochemical impedance spectroscopy

## Abstract

An impedimetric immunosensor for fumonisins was developed based on poly(2,5-dimethoxyaniline)-multi-wall carbon nanotubes doped with palladium telluride quantum dots onto a glassy carbon surface. The composite was assembled by a layer-by-layer method to form a multilayer film of quantum dots (QDs) and poly(2,5-dimethoxyaniline)-multi-wall carbon nanotubes (PDMA-MWCNT). Preparation of the electrochemical immunosensor for fumonisins involved drop-coating of fumonisins antibody onto the composite modified glassy carbon electrode. The electrochemical impedance spectroscopy response of the FB_1_ immunosensor (GCE/PT-PDMA-MWCNT/anti-Fms-BSA) gave a linear range of 7 to 49 ng L^−1^ and the corresponding sensitivity and detection limits were 0.0162 kΩ L ng^−1^ and 0.46 pg L^−1^, respectively, hence the limit of detection of the GCE/PT-PDMA-MWCNT immunosensor for fumonisins in corn certified material was calculated to be 0.014 and 0.011 ppm for FB_1_, and FB_2_ and FB_3_, respectively. These results are lower than those obtained by ELISA, a provisional maximum tolerable daily intake (PMTDI) for fumonisins (the sum of FB_1_, FB_2_, and FB_3_) established by the Joint FAO/WHO expert committee on food additives and contaminants of 2 μg kg^−1^ and the maximum level recommended by the U.S. Food and Drug Administration (FDA) for protection of human consumption (2–4 mg L^−1^).

## 1. Introduction

**Figure 1.**
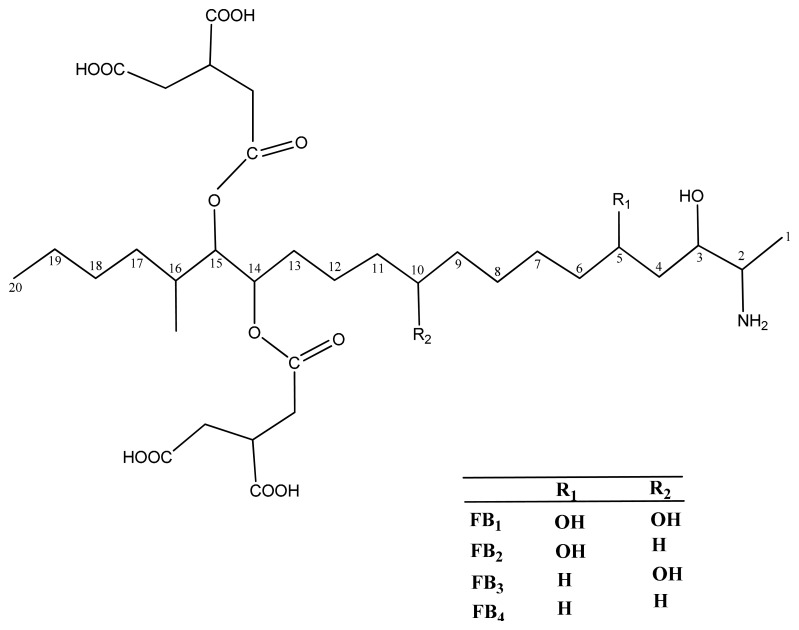
Chemical structure of fumonisins.

In 1996 Pitt defined mycotoxins as fungal metabolites which when ingested, inhaled or absorbed through the skin can cause lowered performance, sickness or death in man or animals, including birds [1,2]. Mycotoxins are an extremely diverse group of low molecular weight biological compounds, and their chemical structures and physical properties vary widely. They are found in different chemical groups (e.g., pyrones, anthraquinones, coumarins, macrolides, steroids and cyclic polypeptides) and the biological conversion products of mycotoxins are also referred to as mycotoxins [3]. Fumonisins represent a group of mycotoxins which were identified and characterized in 1988, and are among the most important mycotoxins concerning food and feed safety and the less studied [4,5]. Their chemical formula is diesters of propane-1,2,3-tricarboxylic acid and either 2-(acetylamino)- or 2-amino-12,16-dimethyl-3,5,10,14,15-pentahydroxycosane [6]. They are produced by a several *Fusarium* species such as *Fusarium verticillioides* (*Fusarium moniliforme*), *Fusarium proliferatum*, and *Fusarium nygamai* [4,7]. Actually over 15 different fumonisins have been discovered and fumonisins B_1_ (FB_1_) and B_2_ (FB_2_) are the major compounds, while the others (FB_3_, FB_4_, FA_1_, FA_2_ and FC_1_) occur in very low concentrations and are less toxic. The structures of some fumonisins are shown in Figure 1.

Fumonisin B_1_ (FB_1_) is the most abundant and toxic of this family of mycotoxins. Generally, corn and corn products are the major commodities which contain important amounts of fumonisins [5]. Although some occurrence has been found in various cereals or food and feed products such as sorghum, rice, wheat, asparagus, cowpeas, maize, millet, farro, black tea, and beer [5,7]. Since 2009, fumonisins have been reported to be found in many other commodities and foods including duck tissue, botanical roots, coffee beans, dried figs, milk, garlic powder, onion powder, spices, traditional African herbal medicines and barley [8]. Fumonisins affect animals in different ways by interfering with sphingolipid metabolism. They have been reported to cause various diseases in animals and humans such as leukoencephalomalacia in horses and rabbits, pulmonary edema and hydrothorax in swine and pigs, liver and kidney toxicity and carcinogenicity and immunosuppression in rats, and esophageal cancer in humans [4,9,10]. In order to protect human consumption and since fumonisins have been involved in probable esophageal cancer hot spots in regions of Transkei (South Africa), China, and northeast Italy, fumonisins have been classified by the U.S. Environmental Protection Agency (EPA) [9] and the International Agency for Research on Cancer (IARC) [5] as 2B carcinogens (possible human carcinogen). Therefore, in 2001, the Joint FAO/WHO expert committee on food additives and contaminants (JECFA) established a provisional maximum tolerable daily intake (PMTDI) for fumonisins (the sum of FB_1_, FB_2_, and FB_3_) of 2 μg kg^−1^ of body weight per day and later the U.S. Food and Drug Administration (FDA) fixed the maximum level of fumonisins to be 2–4 mg L^−1^.

A biosensor is defined as a transducer that incorporates a biological recognition component as the key functional element. It consists of three main components including the biorecognition element, the transducer and the signal display. The interaction of the analyte with the biorecognition element is converted into a measurable signal by the transduction system, which is then converted into a readout. Analytical immunosensors are a subset of biosensors which utilise either an antigen or antibody as the biospecific sensing element. When an antibody is used as molecular recognition element for a specific analyte (antigen) to form a stable complex, the device is then called an immunosensor. Electrochemical transducers are the oldest and most common technique used in biosensing. They offer some advantages such as high specificity, low-detection limits, relative freedom from platform (sensor support) interference and low cost. Meanwhile, they have also some disadvantages including high performance and cost-effectiveness [11]. Electrochemical transducers can be divided into potentiometric, amperometric and impedimetric. Electrochemical immunosensors have been extensively used for the direct detection of antibody-antigen interactions. Various kinds of immunosensors including optical waveguide light mode spectroscopy (OWLS) [12], fluorescent biosensor arrays, electrochemical [13] and impedimetric immunosensors [14] have been developed for clinical and environmental purposes. Their main advantages are that they are non-invasive and require little sample pre-treatment [15]. Due to their high sensitivity, electrochemical techniques including voltammetry and impedance have been successfully used in the detection and determination of various biological compounds. Since, electrochemical immunosensors are concerned with the formation of a recognition complex between the sensing biomaterial and the analyte under investigation in a monolayer or thin-film configuration on an electronic transducer, the formation of a complex on a conductive surface may alter the capacitance and the resistance at the surface-electrolyte interface [16], and this alteration is exploited to determine the concentration of the required analyte. Impedance spectroscopy is a powerful tool for the characterisation of biological thin-films involved in the transduction of biomolecular interactions at electrode surfaces [17], and the kinetics and mechanisms of electron-transfer processes that correspond to the biocatalytic reaction occurring at modified electrodes can be derived from Faradaic impedance spectroscopy (FIS). Many conducting polymers (CP) including polyaniline (PANI), polypyrole (PPy), polycarbazole and polyazines possess acidic or/and basic groups which can be protonated or deprotonated. The strong and reversible influence of these oxidation/reduction, protonation/deprotonation and conformational changes on the electrical and optical properties of conducting polymers make possible their use as transducers or as components of transducers. In this work, electroactive poly(2,5-dimethoxyaniline)-multi-wall carbon nanotubes doped with palladium telluride quantum dots was used as a conductive platform for the preparation of an immunosensor. An impedimetric immunosensor was chosen for this work due to its high stability and antibody-enhanced orientation [14,18].

## 2. Experimental Section

### 2.1. Chemical and Reagents

2,5-Dimethoxyaniline (98%) was purchased from Aldrich (Johannesburg, South Africa) and Fluka (Johannesburg, South Africa. Fumonisins B_1_ (FB_1_) from *Fusarium moniliforme* was received from Sigma-Aldrich, dissolved in methanol at 1 mg mL^−1^ and stored as aliquots in tightly sealed vials at −20 °C. A monoclonal antibody of fumonisin from mouse (catalogue number ABIN346857) was supplied by Antibodies-online, Aachen, Germany. The antibody is lyophilized from 200 μg of protein A purified antibodies and was quoted as specific to fumonisin, immunogen: BSA-Fumonisin, isotype: IgG1/Lambda, cross-reactivity not yet tested. The antibody was also aliquoted and stored at −20 °C until use. Certified corn reference material was purchased from Trilogy^®^ (Washington, MO, USA) and the ELISA kit (Veratox for fumonisin: range of 1 to 6 ppm) was purchased from Neogen Corporation (Lansing, MI, USA). Home grown carbon nanotubes (CNTs; diameter of 40–200 nm and length up to 20 μm; synthesized according to the Ndungu *et al.*, method [19]. Tellurium, powder 99.997% (Te), 3-mercaptopropionic acid 99% (MPA), palladium chloride, sodium borohydrate 98% (NaBH_4_), sodium hydroxide (NaOH), sodium chloride (NaCl), sulfuric acid (H_2_SO_4_), hydrochloric acid (HCl), bovine serum albumin (BSA), absolute ethanol and basic salts including, NaH_2_PO_4_, Na_2_HPO_4_ and KCl used in the preparation of 0.1 M phosphate buffer saline containing 0.1 M KCl at pH 7.4 (PBS) were received from Sigma-Aldrich. Phosphate buffered saline containing KCl and NaCl (10 x PBS) but diluted to 1 x PBS to reach a pH 7.4 with concentration of 0.1 M (PBS) was obtained from Antibodies-online. All electrochemical measurements for fumonisin B_1_ (FB_1_) and other fumonisins (Fms) were carried out in 0.1 M phosphate-buffered saline. All other chemicals were of analytical grade, and deionized water (18.2 MΩ cm) purified by a Milli-Q™ system (Millipore, Johannesburg, South Africa) was used as the reagent water for aqueous solution preparation and analytical grade argon (Afrox, Cape Town, South Africa) was used to degas the system.

### 2.2. Instrumentation

Electrochemistry experiments were carried out with a BASi Epsilon –Ec-ver.2.00.71_XP electrochemistry work station for cyclic voltammetry (CV, BASi, West Lafayette, IN, USA) and electrochemical impedance spectroscopy (EIS) measurements were recorded with a Zahner IM6ex system (ZAHNER-elektrik GmbH & Co. KG, Kronach,, Germany) using electrodes from BioAnalytical systems (BASi, West Lafayette, IN, USA) in a three-electrode electrochemical cell. Impedimetric data and voltammograms for all electrochemical experiments were recorded with a computer interfaced to the Zahner and the BASi Epsilon electrochemical workstation. Glassy carbon electrode (GCE) of area 0.071 cm^2^ and 3 mm of diameter was used as a working electrode. A platinum wire from Sigma-Aldrich and Ag/AgCl electrodes from BAS were used as auxiliary and reference electrodes, respectively. Alumina powders and microcloth pads obtained from Buehler (Lake Bluff, IL, USA) were used for polishing the GCE.

### 2.3. Preparation of Polyanilino-Carbon Nanotubes Doped with Palladium Telluride Quantum Dots

#### 2.3.1. Preparation of PdTe Quantum Dots

3-MPA-capped PdTe quantum dots were directly synthesized according to a procedure that uses palladium chloride salt as metal precursor and hydrogen telluride gas as source of tellurium. Briefly, PdCl_2_ (2.35 mmol) was dissolved in cold water (125 mL) and 3-mercaptopropionic acid (MPA, 5.7 mmol) was added to the solution under stirring, followed by dropwise addition of 1 M NaOH to adjust the pH to 12. Nitrogen gas was then bubbled through the resulting solution for about 45 min after which it was allowed to react with H_2_Te gas as follows: under stirring, H_2_Te gas was generated by the reaction of NaBH_4_ (reducing agent) with Te powder in the presence of injected 0.5 M H_2_SO_4_ (20 mL) under a flow of nitrogen gas for about 25 min. The molar ratio of Pd^2+^:Te^2−^:thiol was 1:0.5:2.4. A change of colour from yellow to orange was observed for the solution which was used as the PdTe semiconductor nanocrystal precursor at this stage. The solution was then kept refluxing under air at 100 °C for 30 min. To prevent post-reaction the solution were kept in the freezer (−20 °C) for further analysis. The 3-MPA-capped PdTe quantum dots modified GCE prepared will be denoted as GCE/PT.

#### 2.3.2. Preparation of Layer by Layer of PDMA-MWCNT with PdTe-3MPA Quantum Dot Modified Glassy Carbon Electrode

Prior to preparation of the platform on the working glassy carbon electrode, the surface of the electrode was preconditioned as follows: the GCE was first polished using 0.3 and 0.05 mm alumina slurries and then rinsed with distilled water. An aliquot (20 μL) of the palladium telluride quantum dots was drop-coated onto the clean and dry glassy carbon electrode surface and dried in open air or under a lamp. The film of material cast on the electrode surface was then gently rinsed with deionized water. This followed by electrodeposition in the solution containing a mixture of 0.1 M of 2,5-dimethoxy-aniline (DMA, 5 mL) and MWCNT (100 μL) prepared in 1.0 M HCl, then degassed with argon gas for 10 min before electropolymerization. The doped 2,5-dimethoxyaniline was polymerized on the surface of GCE/PT by scanning the working electrode potential repeatedly between −200 and +900 mV for 10 cycles at a scan rate of 50 mV/s. The PDMA-MWCNT modified GCE/PT prepared was denoted as GCE/PT-PDMA-MWCNT.

### 2.4. Fumonisins Antibody Immobilisation

Antibody immobilisation of PT-PDMA-MWCNT was achieved by drop coating 20 μL of 0.1 μL μg^−1^ fumonisins antibody and allowing to dry at +4 °C for 24 h in a fridge. After the immobilisation of the antibody, the electrodes were rinsed with PBS in order to remove the excess of physically bound antibody and then BSA (40 μL) was used to block nonspecific binding sites for 2 h at room temperature. The immobilised electrode was denoted as GCE/PT-PDMA-MWCNT/anti-Fms and then rinsed again with PBS, before making electrochemical measurements.

### 2.5. Extraction of Fumonisin Mixture (Fms) from Certified Corn Reference Materials

Extraction was conducted by following the procedure described by Veratox ELISA Kit. Preparation of a ground corn certified reference material involved the weighing out of the sample (5 g) and mixing it with 70% methanol (HPLC grade, 25 mL) together with 30% deionised water (25 mL of 7:3 mixture) in 50 mL centrifuge screw cap vials. The solution was shaken vigorously for 3 min and centrifuged for 15 min at 4500 rpm and the supernatant was extracted through a Whatman filter paper. The filtrate was then collected as a sample for analysis without further preparation. Ten μL of the filtrate was spiked successively in increasing volumes in PBS solution (5 mL) in the electrochemical cell for EIS measurements of Fms.

## 3. Results and Discussion

### 3.1. Optimization of Experimental Conditions

#### 3.1.1. Effect of Antibody Concentration

**Figure 2.**
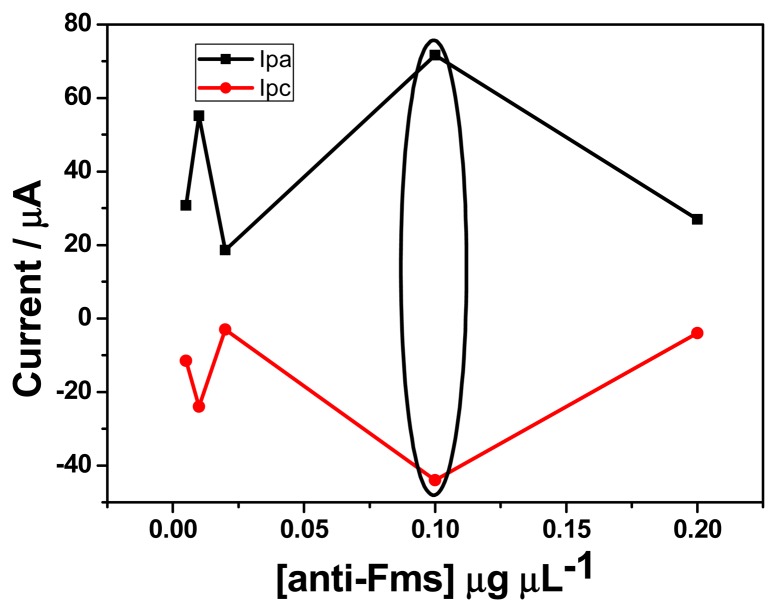
Effect of fumonisin antibody concentration in PBS.

In order to determine the best concentration of fumonisin antibody for the electrochemical response of the immunosensor, different dilutions of fumonisin antibody were prepared (1:5; 1:10; 1:50; 1:100 and 1:200 v/v, corresponding to 0.005, 0.01, 0.02, 0.1 and 0.2 μg μL^−1^). Twenty μL of each concentration was drop coated onto GCE/PT-PDMA-MWCNT and cyclic voltammetry was performed in PBS (pH 7.4) in the potential range of −900 to 900 mV at scan rate of 20 mV s^−1^. As shown in Figure 2, the highest anodic and cathodic peaks current were obtained with 0.1 μg μL^−1^. On the other hand, an experiment was also performed in PBS containing 3 × 10^−5^ μM of FB_1_ (figure not shown) using EIS. The highest value for the charge transfer resistance was obtained with 0.1 μg μL^−1^ compared with other concentrations (0.005, 0.01, 0.02, and 0.2 μg μL^−1^). This result is in agreement with the highest current obtained in cyclic voltammetry in the absence of analyte, thus a concentration of 0.1 μg μL^−1^ was selected for all experiments.

#### 3.1.2. Effect of Temperature

**Figure 3.**
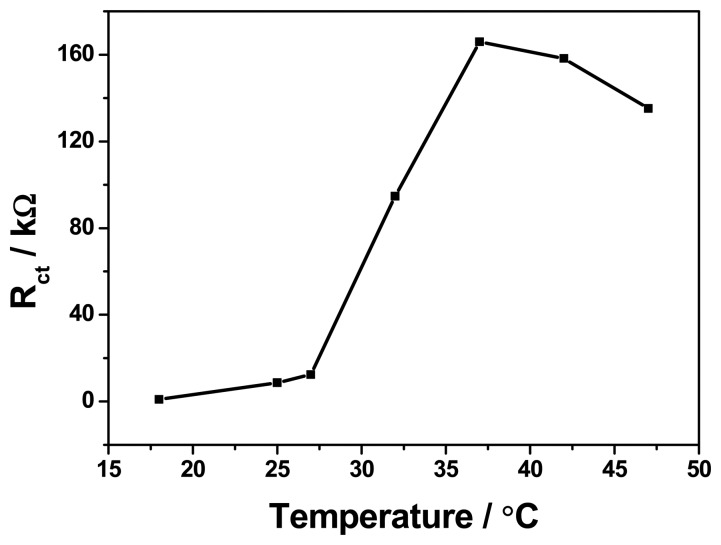
Effect of temperature incubation.

The response of the antigen-antibody reaction depends greatly on the temperature of incubation, thus, effect of temperature incubation on the electrochemical behaviour of the immunosensor was investigated at different temperatures ranging from 15 to 45 °C. As shown in Figure 3, the immunosensor displayed a maximum increase in impedance at 37 °C with incubated antibody solution. Although 37 °C was the best incubation temperature for antibody-antigen reaction, the activity of the immunoprotein is not maintained for a long period of time at this temperature, therefore, the 25 °C (room temperature) was employed as the optimum temperature for practical applications.

#### 3.1.3. Effect of Hysteresis

**Scheme 1.**
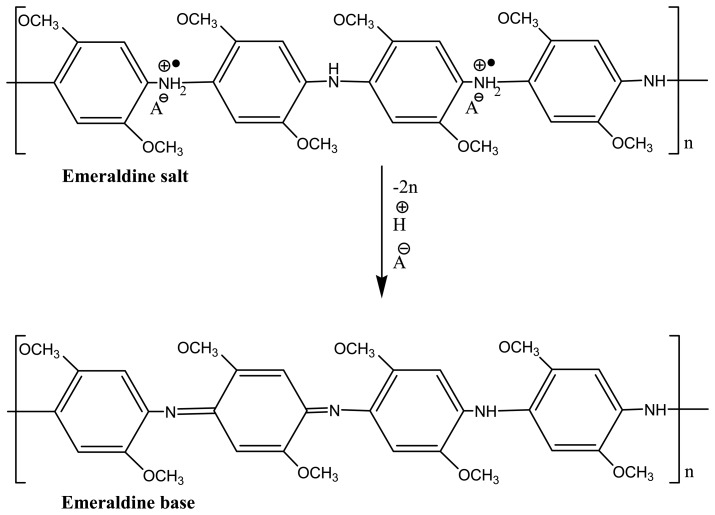
Protonated PDMA emeraldine salt (ES) is deprotonated to emeraldine base (EB) by conditioning in PBS.

Electrochemical synthesis of PDMA and its composites on GCE was done in 1 M HCl as explained previously. The use of acidic medium is necessary to produce polyaniline emeraldine salt which is the most conducting state of aniline [20–22]. To lower the effects of hysteresis, PT-PDMA-MWCNT was conditioned in 0.1 M PBS using cyclic voltammetry [23]. It is well known that the electronic structure of PANI film in its protonated emeraldine salt (ES) form is similar to that of a metal and its conductivity is due to the formation of a polaron band resulting from the proton-induced spin-unpairing mechanism [24]. Thus, in order to convert ES into its semiconducting emeraldine base (EB) as shown in Scheme 1 below, the emeraldine salt (ES) should be treated with a neutral solution, which was phosphate buffer solution (PBS pH 7.4) in our case.

**Figure 4.**
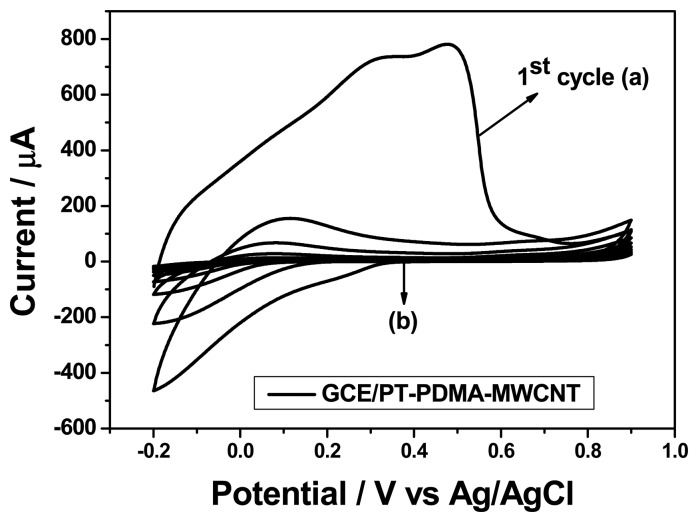
Cyclic voltammograms of GCE/PT-PDMA-MWCNT in PBS: (a) immediately after electrosynthesis and (b) after conditioning (cycling until equilibration of voltammetric current).

A high current was observed in the first cycle (a) compared to the other cycles (b) as shown in Figure 4 for GCE/PT-PDMA-MWCNT. This is attributed to fact that the polymer film was still in its emeraldine salt state and its internal microscopic environment was still acidic. After the first cycle, a decrease in current was observed as the emeraldine salt was converted to emeraldine base. The emeraldine base is known to be an *n*-type semiconductor in neutral PBS. The conductivity of emeraldine salt has been reported to be 1 S cm^−1^ while that of emeraldine base (EB) is between 10^−8^ to 10^−1^ S cm^−1^ [25]. The Fermi level now lies below the conduction band in this semiconductor. The electrochemical potential of the buffer solution determined by the redox potential of the electrolyte solution and the semiconductor is determined by the Fermi level. The charge transfer abilities of the doped PANI semiconductor film depend on the availability of excess charge in the accumulation layer or a depletion layer. In addition, this excess charge on a metallic electrode does lie at the surface, which is not the case for semiconductor electrode. This region referred to as the space charge region, has an associated electrical field. Hence, the interfacial equilibrium between the semiconductor PDMA electrodes and PBS electrolyte solution could be attained when the electrochemical potential of the two phases (PDMA|PBS) became equal.

#### 3.1.4. Choice of Potential

**Figure 5.**
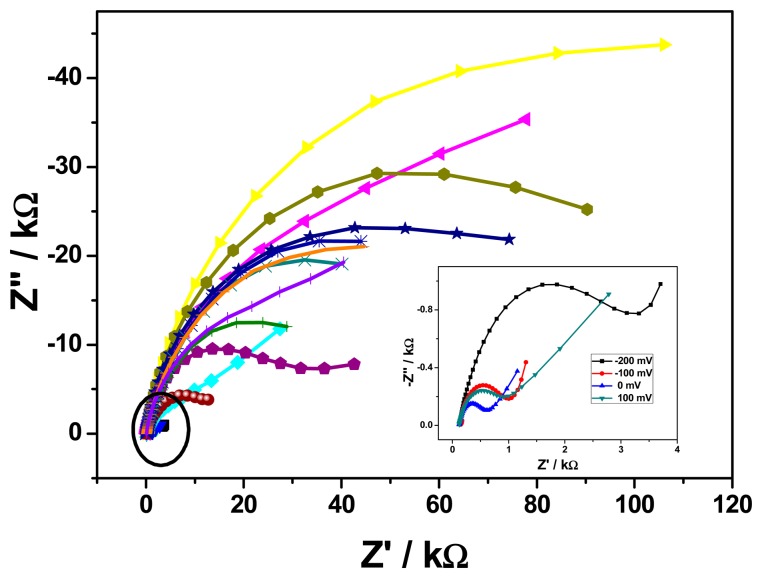
Nyquist plots for GCE/PT-PDMA-MWCNT at the different potential (−800 to 700 mV), performed at 100 mV intervals.

In order to get better potential or best conductive potential, the EIS of buffer-conditioned of GCE/PT-PDMA-MWCNT electrodes was run over a potential range of −800 to +700 mV. A Nyquist plots is shown in Figure 5.

**Figure 6.**
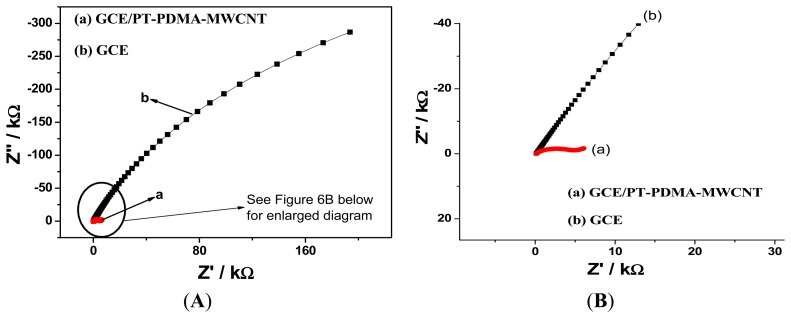
(**A**) Nyquist plots of GCE/PT-PDMA-MWCNT (a) and GCE (b) in PBS at 0 mV; (**B**) Enlarged Nyquist plots of GCE/PT-PDMA-MWCNT (a) and GCE (b) in PBS at 0 mV.

Based on different equivalent circuits used for modelling, and charge transfer resistances (*R*_ct_) obtained, the optimal AC potential was found to be 0 mV, having a smallest value of charge transfer resistance or diameter of semi-circle when compared with the value obtained at −800, −700, −600, −500, −400, −300, −200, −100, +100, +200, +300, +400, +500, +600 and +700 mV, as shown in Figure 5. The potential found in this study is in accordance to those obtained by Muchindu *et al.* [26] and Owino *et al.* [27]. In addition, this zero potential is ideal for the EIS studies since it leads to electronic circuit simplicity and low induced diffusion current. The electrochemical impedance spectroscopy (EIS), is a powerful electroanalytical technique to probe the electron transfer kinetics of modified electrodes [28]. The Nyquist plots of GCE and GCE/PT-PDMA-MWCNT at 0 mV are shown in Figure 6.

The GCE (curve b) shows the characteristics of a diffuse limiting step of the electrochemical process with a high charge transfer resistance as shown by impedimetric parameters (*R*_ct_ = 8.165 × 10^5^ kΩ and *R*_s_ = 127.9 Ω). After being modified with PT-PDMA-MWCNT, the resistance of the film-modified electrode decreased (curve a), indicating a higher electron-transfer at the electrode interface (*R*_ct_ = 2.383 kΩ and *R*_s_ = 125.4 Ω). The drastic drop in the *R*_ct_ value of modified electrode can be attributed to the charge delocalization along the conducting polymer film and to the excellent electrochemical activity of PBS, as well as the good conductivity of the film, which makes the polymer electrode very suitable for charge transfer applications and electrostatic deposition of charged biomolecules.

**Figure 7.**
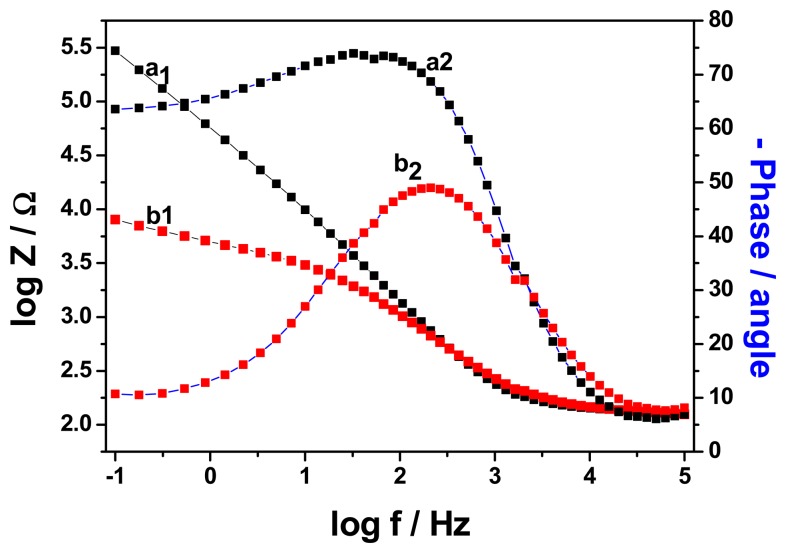
Frequency dependences of the impedance (a_1_and b_1_) and phase shift (a_2_ and b_2_) in the Bode plots of GCE (a_1_ and a_2_) and GCE/PT-PDMA-MWCNT (b_1_ and b_2_) in PBS at 0 mV.

Figure 7 shows the frequency dependence of both the impedance and the phase angle of the unmodified and the modified electrodes. The figure shows an increase in the impedance of the electrodes as frequency decreases. However, the impedance of GCE/PT-PDMA-MWCNT was generally lower than that of GCE over the frequency range studied, which is an indication of improved conductivity of the modified electrode system. At low frequencies, the phase shift was about 10° for the modified electrode and 63.35° for the unmodified electrode. This behaviour of modified electrode is consistent with a one dimensional hopping process as the transfer of the electron from one chain to an adjacent chain is negligible [29]. The phase shift increased with frequency until a characteristic frequency, fc, where the phase shift has a maximum value of 73.43° for GC electrode and 49° for PT-PDMA-MWCNT modified GC electrode. At high frequencies, the phase shift for both, modified and unmodified electrodes have almost the value, 8° and 7°, respectively. The kinetic analysis of the modified and unmodified electrodes showed, the exchange current (*i*_o_) and heterogeneous rate constant (*K*°) for GCE/PT-PDMA-MWCNT are 1.08 × 10^−2^ A and 1.57 × 10^−5^ cm s^−1^, respectively. These values are greater compare to those of GCE, 3.14 × 10^−8^ A and 4.58 × 10^−11^ cm s^−1^, respectively. This result indicated that the modification of the GC electrode with PT-PDMA-MWCNT improved the kinetics at the electrode surface.

### 3.2. Performance of the Immunosensor

**Figure 8.**
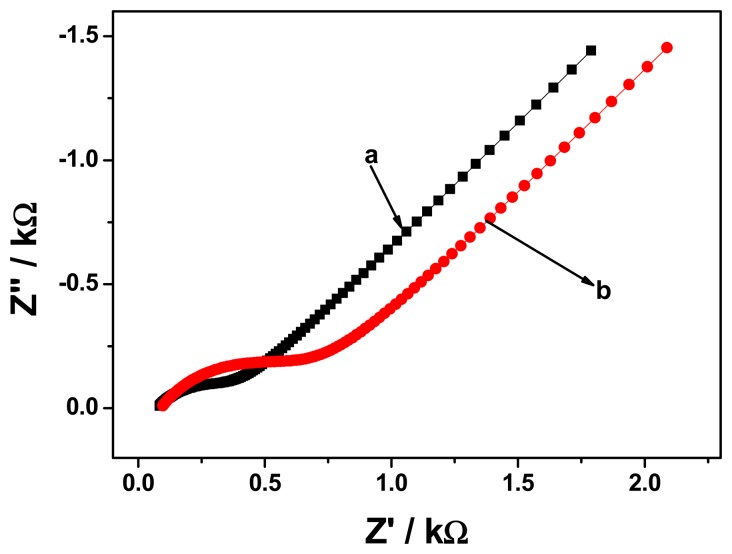
Nyquist plots of GCE/PT-PDMA-MWCNT after antibody immobilisation (curve a) and after immobilisation and blocking with BSA (curve b) in solution of 3 × 10^−5^ μM of FB_1_.

**Figure 9.**
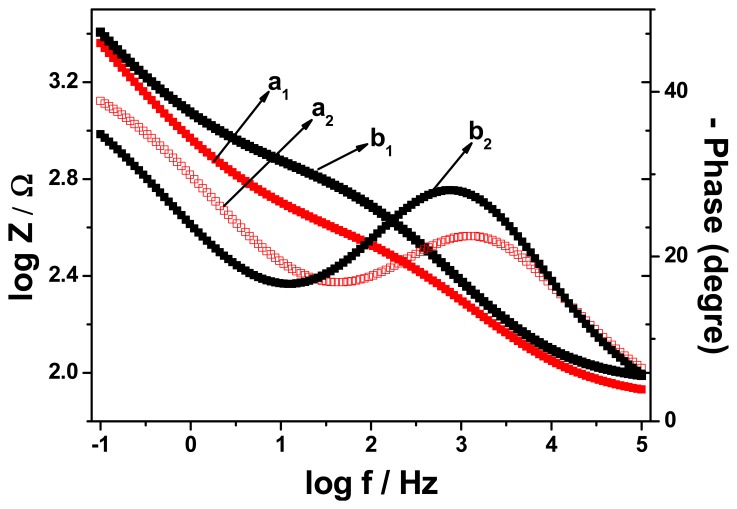
Bode plots of GCE/PT-PDMA-MWCNT after antibody immobilisation (curves a_1_ and a_2_) and after immobilisation and blocking with BSA (curves b_1_ and b_2_) in solution of 3 × 10^−5^ μM of FB_1_.

In order to construct a better immunosensor, electrochemical impedance spectroscopy of GCE/PT-PDMA-MWCNT after antibody immobilisation and after immobilisation and blocking with BSA, was studied in PBS containing 3 × 10^−5^ μM of FB_1_. Figures 8 and 9 show representative Nyquist and bode plots of the electrochemical impedance of GCE/PT-PDMA-MWCNT after antibody immobilisation (curve a) and after immobilisation and blocking with BSA (curve b). In the Nyquist plot, the diameter of the semicircle represents the electron transfer at the electrode. This parameter controls the electron transfer kinetics of the redox-probe at electrode interface, which is relative to the concentration of the analyte. As can be seen in Figure 8, the semicircle diameter of GCE/PT-PDMA-MWCNT after antibody immobilisation is half (with *R*_ct_ = 329.2 Ω) of the GCE/PT-PDMA-MWCNT after antibody immobilisation and blocking with in BSA (*R*_ct_ = 625.8 Ω). The increase in charge transfer resistance after blocking with BSA indicating a higher electron transfer resistance at the electrode. The explanation was that BSA can block possible remaining active sites and further hinder the electron transfer, which confirm that BSA was successfully immobilised on the electrode. The difference observed in *R*_s_ values, 77 Ω and 90 Ω for GCE/PT-PDMA-MWCNT/anti-Fms and GCE/PT-PDMA-MWCNT/anti-Fms-BSA, respectively, is a result of a change in the proximity of the working electrode to the reference electrode [26,30].

**Figure 10.**
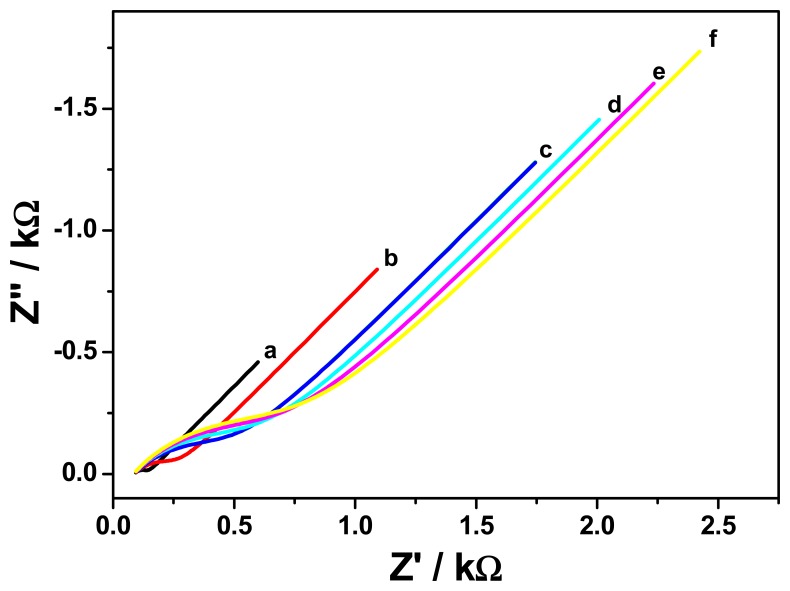
EIS response of FB_1_ immunosensor (GCE/PT-PDMA-MWCNT/anti-Fms-BSA) at different concentrations of 0 (a); 14 (b); 21 (c); 28 (d); 35 (e) and 42 (f) ng L^−1^) in PBS.

The Bode plots (Figure 9) shows the impedance responses (curves a_1_ and b_1_) of the antibody immobilised electrode (a_1_) and after blocking the electrode with BSA (b_1_). It can be seen that the impedance value at a given frequency increased after blocking the electrode with BSA on the antibody immobilised electrode surface in the entire frequency range of interest, indicating that the BSA affects the impedance spectroscopy. The immunoaffinity reaction in the immunosensor frequently involves an irreversible binding of the antigen (Ag) to the binding site(s) of antibodies (Abs). A high level of complementarity is required between a paratope (an Ab's binding site) and the compatible binding region of the Ag (the epitope) in order for the noncovalent interactions resulting in the formation of a stable Ab-Ag complex to occur. The bond that holds the antigen and antibody together in the complex can be compared to ionic bonds, hydrogen bonds, hydrophobic interactions, and van der Waals forces. In addition, impedimetric (capacitance or conductance) immunosensors detect changes in the electric field due to antigen-antibody (Ag-Ab) binding which can change the conductivity on the electrode surface. During EIS measurement, the electrical response is detected due to a periodic small amplitude AC current that has been applied. Thus, an increase in impedimetric transduction is observed due to Ag-Ab binding at the surface of electrically conducting platform. The responses of the GCE/PT-PDMA-MWCNT immunosensor to FB_1_ standard were performed with EIS at different concentrations in 5 mL PBS as shown in Figure 10. The charge transfer resistance (*R*_ct_) was increasing with increase of the analyte concentration. The increase in charge transfer resistance is dependent on FB_1_ concentration and it is due to the decrease in current caused by the insulating properties of the complex formed by the binding of Fms-BSA conjugate and anti-Fms antibody [31]. In addition, since the antigen exists as a dianion (FB_1_^−2^) at neutral pH due to the ionization of carboxyl and hydroxyl groups, the binding of this charged antigen (FB_1_) to the fumonisin immunosensor reduces the conductivity [32].

**Figure 11.**
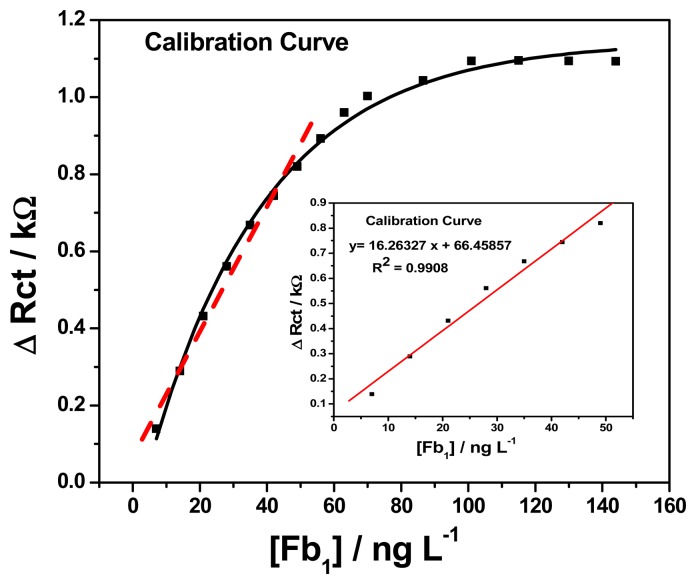
Calibration plot for GCE/PT-PDMA-MWCNT/anti-Fms-BSA immunosensor.

Figure 11 (insert) shows the calibration plot of change in charge transfer resistance *versus* fumonisin B_1_ concentration for GCE/PT-PDMA-MWCNT/anti-Fms-BSA, which exhibited a good linearity, with R^2^ = 0.990 and FB_1_ concentration varying from 7 to 49 ng L^−1^. The corresponding sensitivity and detection limit were 0.0162 kΩ L ng^−1^ and 0.46 pg L^−1^, respectively. In short, the effect of successive addition of FB_1_ is the formation of a layer of antibody-antigen adsorbed over the GCE/PT-PDMA-MWCNT/anti-Fms-BSA. The change in charge transfer resistance (Δ*R*_ct_) increases exponentially with increases of concentration from 7 ng L^−1^ to 144 ng L^−1^ as shown in Figure 11. It can be observed that when Δ*R*_ct_ increases above 63 ng L^−1^ the responses to those changes are no longer linear and tend to stabilize at a constant value beyond the mentioned concentration. The explanation to this behaviour is that when quantities of FB_1_ (analyte) are present in solution, only a portion of the immunosensor (antibody) interacts with FB_1_ (antigen) molecules, increasing in this way the charge transfer resistance due to the interaction between antibody and antigen (FB_1_) as explain earlier. Consequently, as the concentration of FB_1_ increases there are fewer antibody available sites. For that reason, when the surface is saturated of adsorbed molecules, there are no more sites for FB_1_ to be interacted with antibody, so there are no more significative increments in the charge transfer resistance (Δ*R*_ct_). The detection limit was calculated as follows:

(1) $$\frac{3\times SD_{\text{blank}}}{\text{Slope}}$$

with *SD*_(bank)_ being a standard deviation of blank (for eight measurements) and the slope of the calibration plot. The change in charge transfer resistance (Δ*R*_ct_) was obtained using the following equation:

(2) $$\Delta R_{\text{ct}}=\left[R_{\text{ct}}\left({\text{FB}}_{1},X\text{concentration}\right)\right]-\left[R_{\text{ct}}\left(\text{immunosensor},\text{without}\;{\text{FB}}_{1}\right)\right]$$

### 3.3. Stability and Repeatability of Immunosensor

The stability of GCE/PT-PDMA-MWCNT immunosensor was investigated by EIS in PBS containing 4 × 10^−5^ μM of FB_1_. The immunosensor was stored at 4 °C for 5 days and measurements were taken every day. The result shows that the immunosensor retained 53% of its activity. This result indicating relative acceptable stability for the immunosensor is due to the fact that the platform was stable and antibodies were attached firmly onto the surface of electrodes. On the other hand, the stability result obtained may be due to saturation on the electrodes because of the high concentration of FB_1_ used since the bond between antibody and antigen is by affinity.

Meanwhile, the repeatability of immunosensor was evaluated by five successive measurements in the presence of 5 × 10^−5^ μM of FB_1_. The relative standard deviation (RSD) for five parallel measurements with EIS gave 3.9%. Thus, result indicating satisfactory repeatability and the repeatability of the immunosensor was within the experimental error.

### 3.4. Real Sample Analysis

#### 3.4.1. Analysis of Fumonisins Standards and Certified Reference Materials by Enzyme-Linked Immunosorbent Assay (ELISA) Test Kit

**Figure 12.**
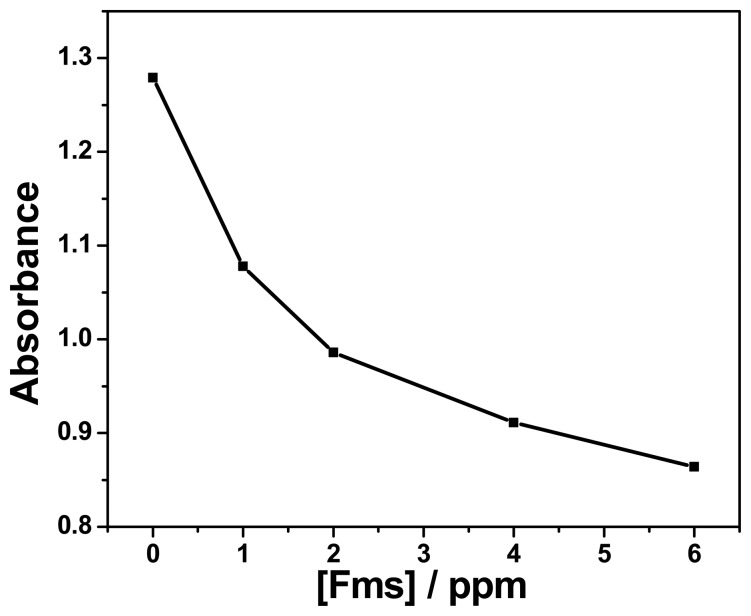
Detection of fumonisin standards curve by enzyme-linked immunosorbent assay (ELISA).

Fumonisin standards and certified reference materials of corn were detected by ELISA for validation of the immunosensors. The concentration range of fumonisin standard in the ELISA test was 1–6 ppm. A standard calibration curve of absorbance *versus* fumonisins concentrations is shown in Figure 12. The sensitivity obtained from the slope of the initial linear part of the graph was calculated to be 0.0305 mg L^−1^ and the limit of detection was 1.97 ppm. These results represent a better parameter in detecting fumonisins and making the use of ELISA appropriate tools for validation of polymeric fumonisin immunosensors. The limit of detection (LOD) for ELISA was calculated using the following equation [9]:

(3) $$\text{LOD}=x{\left[\left(a-d\right)/\left(a-d\right)-3\sigma \right]}^{\overset{-1}{\;}/\underset{b}{\;}}$$

where *a* and *d* are the asymptotic maximum and minimum value of the calibration curves, respectively, *x*: the concentration at the EC_50_ value, b: the slope, σ: standard deviation of blank and EC_50_ is an effective concentration for 50% value.

#### 3.4.2. Real Sample

The GCE/PT-PDMA-MWCNT/anti-Fms-BSA immunosensor was also applied in the detection fumonisins (B_1_, B_2_ and B_3_) extracted from certified reference material corn according to the procedure described in Section 2.5. Table 1 shows the results of GCE/PT-PDMA-MWCNT/anti-Fms-BSA immunosensor to corn certified reference material which are compared to those obtained with ELISA technique as well as the quantity advertised by the vendor.

## 4. Conclusions

In this study we explored the development of an impedimetric fumonisin immunosensor prepared on an electrochemical synthesized PdTe quantum dots–polymer–multi wall carbon nanotubes platform. The modification of the electrode surface and the interaction between fumonisins antibody and fumonisins was studied by impedance spectroscopy. The introduction of biomolecules such as Fms-Ab and BSA and the fumonisin B_1_ standards increases the electron transfer resistance. The obtained electron transfer resistance was used to measure the amount of Fms bound to the immunosensor. It was observed that the antibody layer and antibody-antigen interaction are not conductive and this interaction decreases the electron transfer process on all the developed platforms. The results obtained for the detection of FB_1_ antigen were a positive feedback that can be put to use for trace detection of Fms. The immunosensor had the lowest limit of detection of 0.46 pg L^−1^ with a good sensitivity of 0.0162 kΩ L ng^−1^ for FB_1_ and good repeatability within the experimental error.

## Figures and Tables

**Table 1.** Fumonisins content of corn certified reference material.

**Commodity**	**Mycotoxins**	**GCE/PT-PDMA-MWCNT Immunosensor (ppm)**	**Vendor (ppm)**	**ELISA (ppm)**
Corn	FB_1_ + FB_2_ + FB_3_	0.014 ± 3	-	1.18
Corn	FB_1_	0.014 ± 3	0.01	0.88
Corn	FB_2_	0.011 ± 3	0.01	0.2
Corn	FB_3_	0.011 ± 3	0.01	0.2
